# Emerging role of exosomes in craniofacial and dental applications

**DOI:** 10.7150/thno.48291

**Published:** 2020-07-09

**Authors:** Xin Xing, Shuang Han, Zhi Li, Zubing Li

**Affiliations:** The State Key Laboratory Breeding Base of Basic Science of Stomatology (Hubei-MOST) & Key Laboratory of Oral Biomedicine Ministry of Education, School & Hospital of Stomatology, Wuhan University, Wuhan, China.

**Keywords:** exosome, craniofacial, dental, biomarker, therapy

## Abstract

Exosomes, a specific subgroup of extracellular vesicles that are secreted by cells, have been recognized as important mediators of intercellular communication. They participate in a diverse range of physiological and pathological processes. Given the capability of exosomes to carry molecular cargos and transfer bioactive components, exosome-based disease diagnosis and therapeutics have been extensively studied over the past few decades. Herein, we highlight the emerging applications of exosomes as biomarkers and therapeutic agents in the craniofacial and dental field. Moreover, we discuss the current challenges and future perspectives of exosomes in clinical applications.

## Introduction

Exosomes, which were firstly introduced in the 1980s 1, 2, are nanoscale extracellular lipid bilayer vesicles that are secreted by various cells under physiological and pathological conditions 3. Based on their size and release mechanism, extracellular vesicles (EVs) are classified into three primary types, namely apoptotic bodies, microvesicles, and exosomes. Exosomes are membrane vesicles, with a diameter of 30-150 nm. They are intraluminal vesicles formed by the inward budding of the endosomal membranes during the maturation of multivesicular endosomes 4. The fusion of the multivesicular endosome with the plasma membrane results in the release of individual exosomes. Apoptotic bodies and microvesicles are considered to be larger than 100 nm in size and are released directly from the plasma membrane into extracellular fluid 5. Therefore these two groups will not be further discussed here.

Initially, exosomes were regarded as a simple means for the disposal of unwanted cellular debris. In the past decade, these “waste bags” and their crucial roles in cell communication attracted mounting research attention 6. The nanoscale lipid bilayer exosomes contain various cargos, including proteins, lipids, miRNAs, mRNA, and many other noncoding RNAs. Notably, the presence of these cargo biomolecules depends on their parent cells and organismal status. Through the intercellular transfer of their cargo molecules, exosomes participate in fundamental physiological processes or pathological disorders by regulating the properties of their target cells; their effects can be beneficial or detrimental 7. They are widely distributed throughout bodily fluids, including blood, saliva, breast milk, and urine 8, 9. The functional states of exosome origin cells can be estimated by analyzing the contents of easily accessible exosomes. This approach lays the foundation for exosome-based diagnosis. Except for disease diagnosis, exosomes could be applied as therapeutic tools in various fields, including tissue regeneration, drug delivery, and cancer treatment 10-12. Presently, a total of 167 clinical trials involving exosome-related treatments and diagnoses of various diseases are registered at Clinicaltrials.gov. The speed of the clinical translation of exosome-based diagnosis and therapeutics has far exceeded initial expectations.

In this review, we discuss and summarize up to date and comprehensive literature on the applications of exosomes in the dental and craniofacial field, with particular focus on exosome-based diagnostics and therapeutics. Moreover, the challenges and future development prospects of exosome applications are discussed.

## Exosomes as Diagnostic Biomarkers

**Figure 1:**
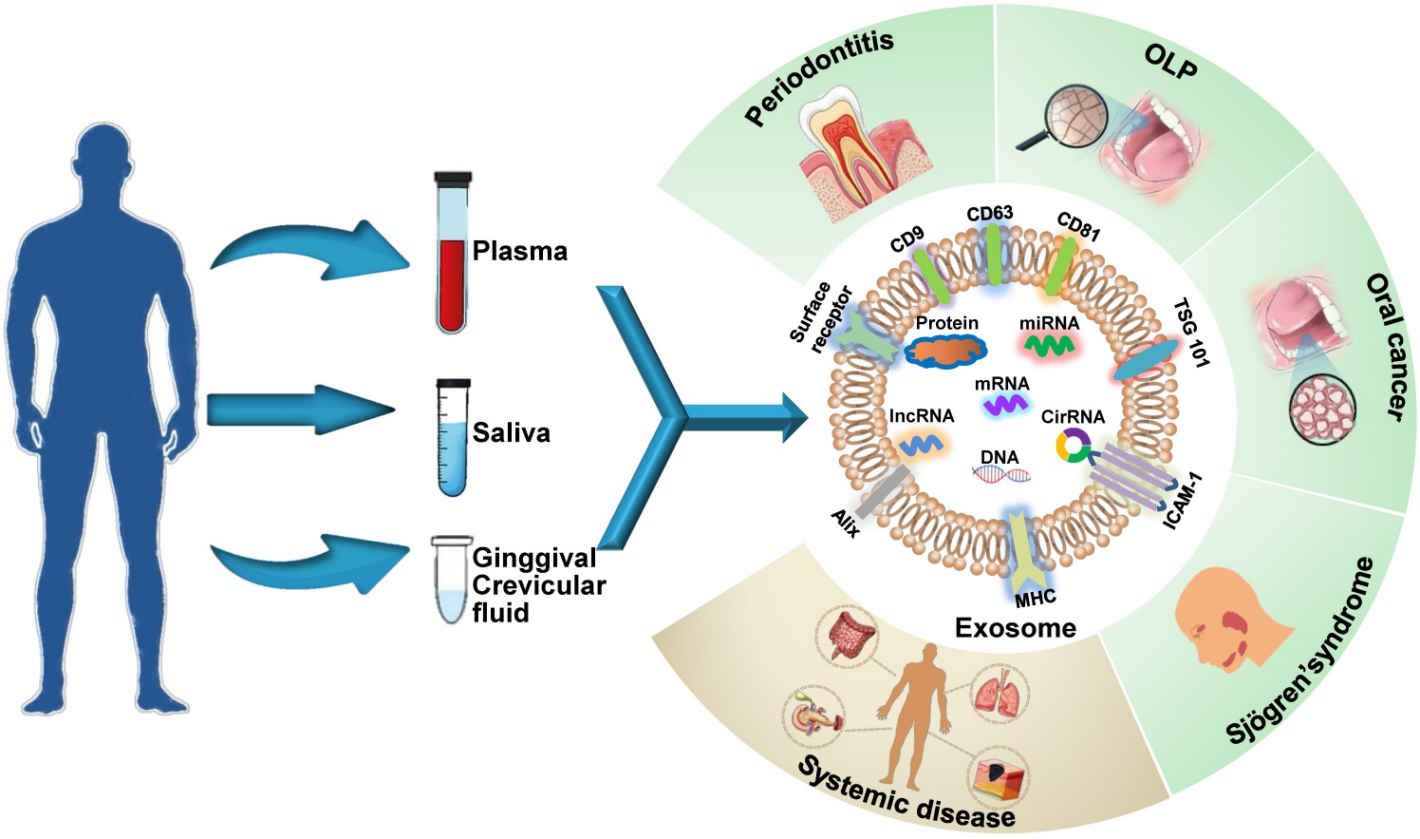
** Schematic presentation of the use of exosomes as diagnostic biomarkers in craniofacial and dental applications.** Exosomes carry multiple proteins and nucleic acids that are specific to the type and condition of their parent cells. The analysis of the concentration, morphology, molecular make-up, and cargos of exosomes circulating in bodily fluids reflects parental cell status and provides insights into disease diagnosis. Exosomes have been shown to act as potential diagnostic biomarkers for periodontitis, oral lichen planus (OLP), oral cancers, and Sjögren's syndrome in the craniofacial and dental field. Additionally, systemic diseases can be diagnosed with the help of exosomes derived from oral liquid.

Exosomes are ideal non-invasive biomarkers for disease diagnosis. They exist extensively in various bodily fluids, their molecular contents are specific to their parental cell type, and their levels of components depend primarily on the functional states of these cells, *i.e.*, whether the cells are in normal physiological state or a pathological state, such as oxidative stress, transformation, apoptosis and abnormal division 13. Therefore, an analysis of exosomal cargos circulating in bodily fluids reflects the altered state of parental cells and provides insights into the diagnosis of systemic and oral diseases (**Figure 1**). Exosome biomarkers for craniofacial and dental diseases are summarized in** Table 1**. The potential oral exosome biomarkers detected in systemic diseases are shown in **Table 2**.

### Exosome biomarkers for craniofacial and dental diseases

#### Periodontitis

Periodontitis is defined as a chronic inflammatory disease that is initiated by the accumulation of microbial plaque and characterized by the progressive destruction of tooth-supporting tissues 14. Periodontitis has a high global prevalence and has become a major public health concern. One of the goals of research on periodontology is the development of high-impact diagnostic biomarkers that have a considerable effect on clinical decision-making, patient outcomes, and healthcare providers 15. This could be attained through the application of protein-containing exosomes. A previous study indicated that reduced salivary levels of CD9/CD81 exosomes are associated with the pathogenesis of the periodontal disease 16. In another study comparing the differences between salivary exosomal proteins in young adults with severe periodontitis and healthy individuals through mass spectrometry (MS) and gene ontology analysis, 26 immune-related proteins were unique to severe periodontitis 17. Besides, exosome-associated nucleic acids could act as potential biomarkers of periodontal disease status. PD-L1 plays an essential role in various cancers and inflammation-etiologic diseases, including periodontitis 18, 19. PD-L1 mRNA in salivary exosomes is enriched in periodontitis, and its level is associated with the severity of periodontitis 20. A recent study revealed three significantly elevated miRNAs (hsa-miR-140-5p, hsa-miR-146a-5p, and hsa-miR-628-5p) in only salivary exosomes in periodontitis patients but not in healthy controls 21. In an additional exploratory study, Chaparro *et al.* detected the total concentration of EVs in gingival crevicular fluid (GCF) and saliva samples from patients with healthy gums/gingivitis or periodontitis. The authors did not detect significant differences in salivary EVs but found significant increases in the total concentration of medium/large EVs in GCF 22. Since GCF consists of serum and locally generated components, including tissue breakdown products, inflammatory mediators, and antibodies in response to oral microorganisms, we speculate that there could be significant differences in GCF exosome levels between periodontitis patients and healthy individuals. However, ascertaining this is experimentally difficult owing to the limited available volume of GCF (microliter level), which could be partially responsible for the lack of literature in this area. Unlike GCF, which is easily contaminated with saliva, blood, and plaque, saliva is a more desirable source of exosome for periodontitis diagnosis. Moreover and notably, the efficacy of plasma exosome biomarkers for periodontitis has not been studied. Therefore, these need further investigations.

#### Oral lichen planus

Oral lichen planus (OLP) is a chronic immune-mediated inflammatory disease of the oral mucosal characterized by various clinical manifestations with keratotic or erythematous and ulcerative lesions 23.

The WHO categorizes OLP as an oral potentially malignant disorder (OPMD) given its malignant tendency, unclear etiology and the lack of a unified therapy 24. A recent study indicated that exosomes are involved in the pathogenesis of OLP 25. Can exosomes be a valuable tool for the diagnosis of OLP? A comparison of salivary exosomal miRNA from 16 patients with OLP and 8 healthy controls revealed that miR-4484 is significantly upregulated in patients with OLP 26. In addition to salivary exosomes, circulating plasma exosomes could serve as potential diagnostic biomarkers for OLP. Peng *et al.* compared the exosomal miRNA profiles isolated from the plasma of patients with OLP with those of healthy individuals by miRNA array analysis. They discovered that circulating exosomal miR-34a-5p is significantly upregulated in patients with OLP and positively correlated with the severity of OLP 27. In general, a biopsy is recommended for definite OLP diagnosis. The above reports suggest that exosome biomarkers are expected to be a superior alternative for the diagnosis of OLP. However, it is difficult to state which one could serve as the most effective biomarker for OLP. The plasma exosomal miR-34a-5p seems to have a significant reference value beacause of its direct association with OLP severity. In contrast, salivary exosomal miR-4484 has distinctive advantages over plasma because saliva sampling is simple, non-invasive, with minimal training requirements compared with blood sampling. Future research should aim at elucidating salivary exosomal biomarkers that are positively correlated with OLP severity as an optimal tool for diagnosis.

#### Oral cancer

Oral cancer is preventable and curable in its early stages. However, considerable cases of oral squamous cell carcinomas (OSCC) are not diagnosed until progressed stages, which are associated with poor therapeutic responsiveness and prognosis 28. Generally, cancer diagnostics rely on tissue biopsies. Nowadays, endeavors have been made to discover novel, non-invasive methods for cancer diagnosis. For instance, liquid biopsy based on the detection of circulating tumor cells (CTCs), circulating tumor DNA (ctDNA) and circulating tumor RNA (ctRNA), and exosomes 29. In squamous cell carcinomas, exosomes have been shown to be crucial components in the tumor microenvironment, suggesting their significance in tumorigenesis, tumor invasion, and metastasis 30. Growing research evidence shows that the characteristics of exosomal morphology, proteins (surface and cargo), and miRNAs serve as potential biomarkers for the diagnosis of OSCC. Sharma *et al.* and Zlotogorski *et al.* attempted to perform atomic force microscopy on exosomes collected from saliva and reported that the morphological features of exosomes differ between patients with oral cancer and healthy individuals 31, 32. Similarly, fourier-transform infrared spectroscopy coupled with computational-aided discriminating analysis was used to assess the diagnostic potential of salivary exosomes from oral cancer patients and healthy individuals. The results of this analysis showed that oral cancer exosomes can be accurately differentiated from their benign counterparts by detecting subtle changes in the conformations of proteins, lipids and nucleic acids 33. The expression of exosomal surface proteins, including CD63, CD9, and CD81, is moreover different in salivary exosomes from patients with oral cancer and healthy individuals in the above-mentioned study by Sharma *et al.* and Zlotogorski *et al.* 31, 32. Besides, a pilot clinical study by Rodriguez *et al.* evaluated the relationship between CD63- and CAV1-positive exosome levels in patients with OSCC before and after surgical treatment and correlated this relationship with overall survival. They found that CD63-positive exosome levels have decreased after surgery, whereas CAV-1 levels have increased most likely due to postsurgery inflammatory response 34. Examining exosomal cargo proteins through proteomic analysis provides a useful diagnostic tool for detecting malignant changes in oral cancers. A study involving quantitative proteomics analysis of serum exosomes in OSCC patients with lymph node metastasis (LNM) identified ApoA1, CXCL7, PF4V1, and F13A1 as potential diagnostic biomarkers but not prognostic biosignatures 35. Research evidence indicated circulating PD-L1 on the surfaces of exosomes isolated from plasma is a useful metric that is associated with disease progression in patients with OSCC 36. Additionally, laminin-332 levels in plasma exosomes from OSCC patients with LNM are markedly higher compared with OSCC patients without lymphatic metastasis; implying that plasma exosomal laminin-332 is a potential and non-invasive biomarker for the detection of lymph node metastasis in OSCC 37. Winck *et al.* measured salivary exosomal proteins of OSCC patients through liquid chromatography-tandem MS (LC-MS/MS) analyses. Through bioinformatics analysis, the authors obtained a priority list of 139 proteins identified from salivary exosomes in patients with OSCC and healthy controls. Statistical analysis revealed that 8 proteins are differentially expressed between the two groups; these proteins may serve as biomarkers of oral cancer 38. Furthermore, exosomal miRNAs, which act as regulatory gatekeepers of coding genes, are potential minimally invasive diagnostic biomarkers that could be used to screen for oral cancer. Rabinowits and colleagues comparatively analyzed miRNA expression in the benign and malignant tissues and plasma of patients with tongue cancer. They found that 16 miRNAs are differentially expressed between tumors and their matched benign tissue. Amongst these miRNAs, 9 upregulated and 7 downregulated miRNAs can be found in circulating exosomes 39. In addition to plasma miRNAs, the application of salivary exosomal miRNAs as diagnostic biomarkers for oral cancer has been extensively studied. In a study comparing the miRNA content of exosomes from the saliva of patients and healthy controls, Gai *et al.* showed that miR-302b-3p and miR-517b-3p are expressed only in OSCC patients and two other miRNAs, miR-512-3p and miR-412-3p, are upregulated in OSCC compared with the healthy individuals 40. Moreover, He *et al.* demonstrated that oral cancer-derived salivary exosomal miR-24-3p may also serve as a potential detective biomarker for OSCC screening 41. Although numerous studies reveal the significant association of exosome biomarkers with oral cancers, the results of these individual studies show poor concordance. Inconsistent isolation strategies of exosomes and stage diversity of cancers may contribute to this mismatch. Additionally, compared with other cancer types for which various diagnostic tests are registered according to Clinicaltrials.gov, the application of exosomal biomarkers in the diagnosis of oral cancer in clinical settings remains absent and requires further research for effective implementation.

#### Sjögren's syndrome

Sjögren's syndrome (SS) is a chronic autoimmune disease that is characterized by lymphocyte infiltration and inflammation in the exocrine glands, particularly the salivary and lacrimal. The disease causes oral and ocular dryness (xerostomia and keratoconjunctivitis sicca) 42. Numerous SS biomarkers have been identified in saliva, tears, and plasma 43. However, research on the application of exosomal biomarkers in SS diagnosis is limited. Michael *et al.* studied salivary exosomal miRNAs using TaqMan quantitative PCR and miRNA microarrays in a patient with SS and healthy individuals 44. Although the author presented their obtained miRNA patterns only as a proof of concept without drawing any disease-specific conclusions, they provided the first report describing the correlation between salivary exosomes and SS. Aqrawi *et al.* performed a proteomic analysis of EVs isolated from the saliva and tears of patients with SS by using liquid chromatography-mass spectrometry (LC-MS). They found that dozens of proteins are significantly upregulated in the salivary EVs of patients with SS compared with the control group. Only 2 proteins from tears are upregulated in patients with SS because of the low tear fluid volumes collected 45. Overall, saliva and tear sample collection is an example of a non-invasive sampling method. Besides the two studies above, additional evidence, such as plasma-derived exosomes, could validate potential exosomal biomarkers for SS. Nonetheless, identifying biomarkers in tears and saliva is desirable since their sampling is manageable, inexpensive, and non-invasive. The possibility of combining salivary and tear exosomal cargos might provide a more precise detection approach for SS; therefore, future research should be focused on this.

### Oral exosome biomarkers for systemic diseases

The role of exosomal biomarkers in oral disease diagnosis is an example of the current applications of exosomes in dentistry. Exosomes isolated from oral fluids can additionally serve as effective biomarkers for systemic disease diagnosis. A previous study revealed high levels of PSMA7 in salivary exosomes in patients with inflammatory bowel disease, suggesting that PSMA7 is a promising biomarker alternative to colonoscopy 46. Machida *et al.* utilised samples from 13 elderly individuals and 15 young healthy volunteers to examine the correlation between salivary exosomal miRNAs and ageing. Through microarray analysis and real-time PCR validation, they identified miR-24-3p as a novel candidate biomarker of ageing 47. Besides oral cancers, salivary exosomes have been used as diagnostic biomarkers in other cancer types, such as pancreatic cancer, pancreatobiliary tract cancer, lung cancer, and melanomas 48-52. Monteiro *et al.*, in a pilot study, demonstrated significantly different concentrations of EVs in the GCF between women with gestational diabetes mellitus and normoglycemic pregnant women; hence, they could be used as an early biomarker for the prediction of gestational diabetes mellitus in pregnant women 53.

Collectively, exosomal morphology, counts, and the levels of exosome-incorporated contents reflect the pathological state of the disease. Exosomes protect their cargos, making them more stable in biological fluids and reliable biomarkers compared to freely circulating biomarkers. These features, as well as their extensive availability in various bodily fluids, make them promising candidates as diagnostic biomarkers of disease. Among all the parental exosome biofluids, the collection of saliva constitutes the most commonly recommended approach for prospective clinical applications based on the advantages discussed earlier. Moreover, the limited sample size and methodological differences in exosome enrichment in the current studies could result in biased estimates and inconsistent results. Therefore, large-scale, prospective multicenter preclinical studies and clinical trials under well-defined conditions (*e.g.*, uniform purification method, standardized detection procedure, and specific pathological stage of disease) are prerequisites for the application of exosomes as precise diagnostic tools in clinical practice. Besides, to achieve higher sensitivity and specificity of exosome-based diagnostics, a combinatorial and multicomponent approach, including combinations of multiple exosomal markers (*e.g.*, nucleic acids in combination with proteins) should also be considered.

## Exosome-based therapeutics for craniofacial and dental applications

**Figure 2:**
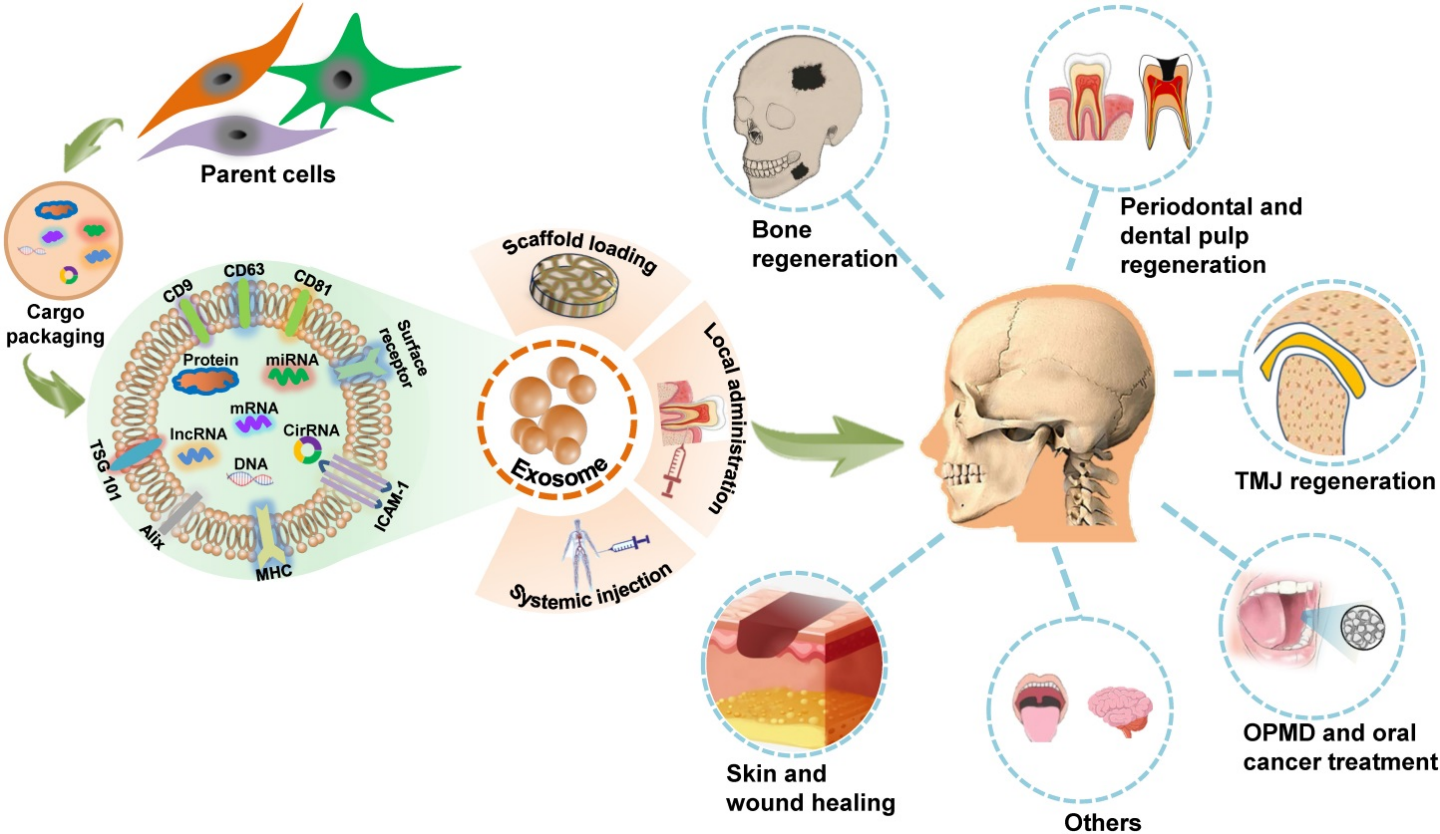
** Schematic presentation of exosome-based therapeutic applications in the craniofacial and dental field.** Exosomes are secreted by many types of cells and modulate biofunctions by conveying unique signals obtained from their parental cells to recipient cells. Through scaffold loading or application *via* local administration or systemic injection, exosomes can function as a novel potential therapeutic tool for regenerating the craniofacial bone, the temporomandibular joint (TMJ), the skin, the periodontium and dental pulp; treating oral potentially malignant disorders (OPMD), oral cancer, and several other craniofacial and dental diseases.

The innate attributes of exosomes indicate that they could be applied in the design of potential therapeutic agents. The nanoscale size, excellent immunocompatibility, rapid endocytosis, nontoxicity, stability, and accessibility to biological barriers of exosomes render them as novel cell-free therapy agents with attractive advantages over their parent cells in disease treatment 54, 55. There is increasing research evidence regarding the *in vitro* exploration of exosome biology and utility in controlling cell activity. The direct applications of exosomes in living organisms, including animal models, or clinical trials, provide an easy-to-access and convincing paradigm for exosome-based therapies. The role of MSCs-derived exosomes in craniofacial tissue engineering and regeneration has been summarized in detail in a previous review 56. Here, we provide a broad overview of *in vivo* applications of exosomes as potential cell-free therapeutic agents in the regeneration of craniofacial bone, skin, temporomandibular joint (TMJ), periodontal tissue and dental pulp, as well as the treatment of oral cancer, OPMD, and other craniofacial and dental diseases (**Figure 2**). A list of studies focusing on *in vivo* exosome-based therapeutic applications in the craniofacial and dental field is provided in **Table 3**.

### Craniofacial bone regeneration

Craniofacial bone defects after trauma, infection, tumor resection and congenital deformities lead to different degrees of deformity and dysfunction in patients. The traditional clinical approaches for repair involve autologous, allogeneic bone grafting, and distraction osteogenesis, which may provide positive results but suffer from shortcomings, including donor site morbidity, immune complications, and cosmetic concerns 57. Bone tissue engineering has emerged as a promising solution to overcome these shortcomings with the controlled application of cells, combined with biocompatible materials, for therapy 58. Angiogenesis and osteogenesis are critical stages in bone regeneration 59. Numerous *in vitro* studies have demonstrated that exosomes act in a paracrine manner to regulate osteogenesis and angiogenesis of recipient cells. Mesenchymal stem cells (MSCs) are currently the most established promising parental sources of exosomes for tissue engineering and regeneration. Exosomes derived from human-induced pluripotent stem cell-derived MSCs (hiPS-MSCs), human adipose MSCs (hADSCs), and human perivascular stem cells (hPSCs) induce naive stem cells into to an osteogenic linage 60-63. Additionally, MSCs-derived exosomes can be used as biomimetic tools to regulate osteoblast proliferation and activity directly 64, 65. Angiogenesis, which provides nutrition and oxygen to the surrounding cells, is a vital step in bone healing. Exosomes derived from multiple types of cells, including the bone marrow-derived MSCs (BMSCs) 66, 67, ADSCs 68-70, human placenta-derived MSCs (hP-MSCs) 71, 72, periodontal ligament stem cells (PDLSCs) 73 promote *in vitro* proliferation, migration, and tube formation of endothelial cells.

In addition to MSCs, exosomes harvested from other cells are potential pro-osteogenic and pro-angiogenic factors. Exosomes from mineralizing osteoblast cells 74, myoblasts 75, periodontal ligament fibroblasts 76, monocytes 77 and macrophages 78 activate osteogenic differentiation in cell culture.

Several *in vitro* studies have also demonstrated that exosomes derived from skeletal muscle 79 and leukemia 80 regulate endothelial cell function and stimulate angiogenesis.

**Figure 3:**
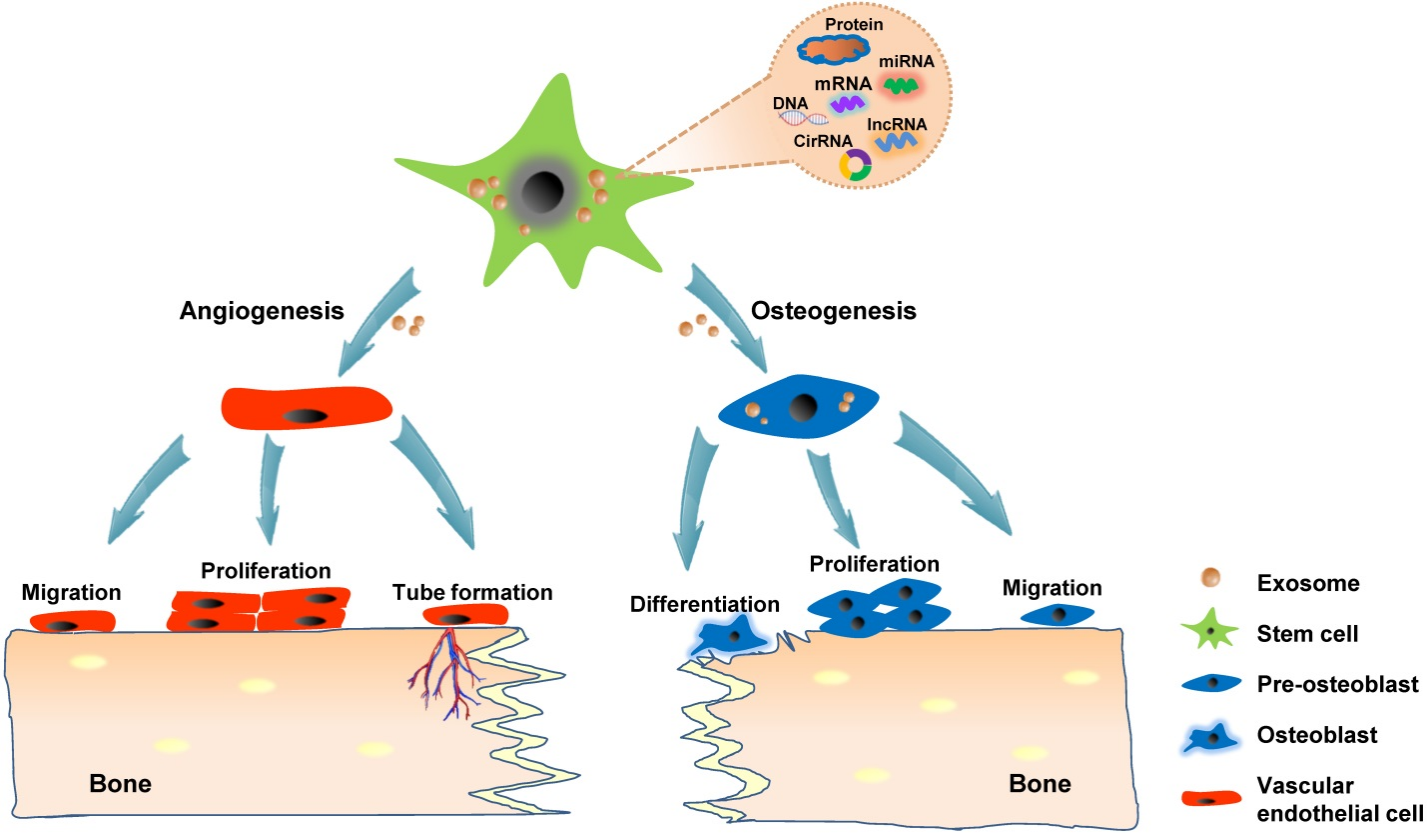
** Exosomes promote bone regeneration through the regulation of angiogenesis and osteogenesis.** Stem cells-derived exosomes convey functional cargos, including various proteins and nucleic acids, into the recipient cells existing in the bone microenvironment. The angiogenesis mechanism involves the activation of the vascular endothelial cells, resulting in migration, proliferation, and the formation of new blood vessels. The potential exosomal osteogenic ability possibly stimulates naive stem cells into to an osteogenic linage or directly regulating osteoblast.

Although numerous *in vitro* studies have indicated the osteogenic and angiogenic capacity of exosomes, direct *in situ* application in animal models is highly convincing and intuitive for proving the effect of exosomes on bone regeneration. The calvarial bone defect model is the most widely used animal model for studying *in vivo* bone regeneration potential of exosomes in craniofacial and dental applications. The strategies for loading exosomes derived from hiPS-MSCs and hADSCs into β-tricalcium phosphate (β-TCP) and polydopamine-coating poly (lactic-co-glycolic acid) (PLGA) scaffolds, respectively have resulted in successful calvarial bone formation 60, 62, 81. Modified exosomes moreover exhibit potential in stimulating bone regeneration; for instance, the patching of hydrogel containing exosomes harvested from miR-375-overexpressing hADSCs over calvarial wounds results in significantly accelerated healing 61. Liang *et al.* showed that loading exosomes derived from dimethyloxaloylglycine-stimulated human bone marrow MSCs into porous hydroxyapatite scaffolds improve bone healing at six weeks after implantation into the critical-sized calvarial defects 67. In addition to the incorporation into a delivery system, exosomes could be applied through direct injection. Recently, exosomes derived from hPSCs were percutaneously injected into the tissue directly overlying a mouse calvarial bone defect, which accelerated bone defect healing 63. Alveolar bone defect models have also been shown as suitable experimental models for verifying the role of exosomes in craniofacial and dental bone regeneration. Wu *et al.* indicated that exosomes secreted *via* stem cells from human exfoliated deciduous teeth (SHEDs) enhance osteogenesis and angiogenesis through the AMPK signaling pathway. By using a rat model of alveolar bone defects, they showed that exosome-loaded β-TCP scaffolds significantly promote bone formation compared with β-TCP or the control treatment 82. Watanabe *et al.* created a bisphosphonate-related osteonecrosis of the jaw (BRONJ) model by administering zoledronic acid to rats and extracting the teeth. Their results indicated that the administration of MSCs-derived EVs prevents senescence of cells involved in wound healing and the spread of chronic inflammation around senescent cells, thereby promoting angiogenesis and bone regeneration and preventing BRONJ 83. Collectively, exosomes, especially stem cells-derived exosomes, could be potentially applied in treating various bone diseases and promoting bone regeneration because of their proangiogenesis ability and stimulatory effects on osteogenic cells (**Figure 3**).

### TMJ regeneration

TMJ is a complex articulation covered by dense fibrocartilage formed between the mandibular condyle and the temporal bone. TMJ diseases have attracted mounting research interests in regenerative strategies that combine stem cells, scaffolds, and bioactive molecules, including exosomes 84. A previous study indicated that plasma-derived exosomes loaded with miR-140 induce BMSCs differentiation into chondrocytes 85. Further, Luo *et al.* showed that miR-100-5p-carrying exosomes derived from SHEDs suppress inflammation in TMJ chondrocytes by activating the mTOR signaling pathways 86. These *in vitro* exosome treatments had positive outcomes and revealed exosomes as potential therapeutic agents for in joint and cartilage repair. Indeed, the various studies that yielded these results were conducted in animal models. However, most studies on the application of exosomes in joint and cartilage regeneration *in vivo* focused on arthritic knees 87. In a recent study, human embryonic stem cell-derived exosomes injected into the compartment of the TMJ in a rat model with monosodium-iodoacetate-induced TMJ osteoarthritis promoted TMJ repair and regeneration by regulating inflammatory responses and healing condylar cartilage and subchondral bone 88. To our knowledge, this is the first *in vivo* study demonstrating the translational potential of an exosome-based therapy for TMJ repair and regeneration. The anatomical, structural, and functional regeneration of TMJ is highly challenging and specific owing to its uniqueness and complexity. Additional research evidence for the appropriate animal and human models is needed to rationalize the application of exosomes in the regeneration of TMJ structures, including the cartilage, subchondral bone, and even the TMJ disc.

### Periodontal and dental pulp regeneration

The periodontium is a hierarchically organized tissue that consists of the gingiva, periodontal ligament, cementum, and the alveolar bone. It provides physical and mechanical support to the teeth. The ultimate objective of periodontal treatment is the regeneration of periodontium, which involves the functional reattachment of the periodontal ligament to the new cementum and the alveolar bone 89. Novel approaches, such as biomaterials and cell-based therapy for periodontal regeneration, have been explored. Although these strategies have shown positive outcomes, they possess the challenges of maintaining cell vitality, immune compatibility, and safety. Exosomes have emerged as a cell-free therapeutic approach with low immunogenicity and increased safety, given their endogenous origins. *In vitro* studies have shown that exosomes involved in inflammatory signal transfer and periodontitis progression, which may provide therapeutic targets for periodontal regeneration 76, 90, 91. Royo *et al.* demonstrated that exosomes derived from PDLSCs regulate angiogenesis *via* the exosome-mediated transfer of miR-17-5p-targeted VEGFA 73. Additionally, SHEDs-derived exosomes enhance PDLSCs osteogenic differentiation partly due to the presence of Wnt3a and BMP2 in the exosomes. This effect provides new insights into the therapeutic use of SHEDs exosomes in treating periodontitis-induced bone defects 92. Mohammed *et al.* firstly investigated the therapeutic effect of exosomes in periodontal regeneration with animal models 93. They injected ADSCs exosomes locally into pockets in a ligature-induced periodontitis rat model. Their results showed remarkable new periodontal tissue formation and provided direct support for the potential application of exosomes for periodontal regeneration. Chew *et al.* reported another study that further verified the *in vivo* periodontal application of exosomes. This report demonstrated that MSCs exosomes enhance periodontal regeneration possibly by increasing periodontal ligament cell migration and proliferation and suggested that MSCs exosomes are an available ready-to-use and cell-free therapy for periodontal defects 94. Notably, according to Clinicaltrials.gov, a human clinical trial (NCT04270006) entitled “Effect of adipose-derived stem cells exosomes as an adjunctive therapy to scaling and root planning in the treatment of periodontitis” is being conducted by an Egyptian researcher. To our knowledge, this is the first and only clinical trial involving the application of exosomes in the craniofacial and dental field.

The dental pulp is richly vascularized and innervated tissue that maintains a wide range of biological and physiological functions, including responding to bacterial insult and injury, providing neuronal sensitivity and transmitting mechanical stimuli for repair and regeneration 95. Infection or necrosis of dental pulp constitutes the most common endodontic disease. The typical treatment of pulp diseases involves root canal therapy to remove diseased dental pulp tissue, followed by filling the root canal with inorganic material. The loss of dental pulp tissue results in loss of tooth vitality. Tissue engineering approaches, including stem cell-mediated functional therapy aimed at regenerating dental pulp, can readily address this issue 96. Recently, the use of exosomes as tools in regenerative medicine has gained prominence. Exosomes derived from dental pulp stem cells (DPSCs) have been shown to suppress inflammation, reduced edema, and promote angiogenesis 97, 98. Moreover, stem cells derived from the dental pulp of human exfoliated deciduous teeth have unique anti-apoptotic and neurogenic properties 99. Ivica *et al.* revealed that exosomes secreted by SHEDs activate the recruitment and proliferation of human MSCs 100. Similarly, Lim and colleagues reported that exosomes derived from human DPSCs under odontogenic conditions promote the odontogenic differentiation of human DPSCs through the TGFβ1/smads signaling pathway *via* the transfer of microRNAs 101. Schwann cells are an essential cellular source of dental MSCs that migrate to injured sites and differentiate into odontoblasts and dental pulp cells 102. Moreover, Schwann cells are nerve-associated glial cells capable of axonal regeneration and reconnection establishment after peripheral nerve injury 103. A recent study showed that exosomes from hDPSCs, particularly from lipopolysaccharide-preconditioned hDPSCs, promote the proliferation, migration, and odontogenic differentiation of Schwann cells 104. The author declared that their findings might provide new insights into the regulatory capability of exosomes from hDPSCs for Schwann cells involved in pulp regeneration. The above studies have provided only *in vitro* evidence for exosome-mediated dental pulp regeneration. However, little information is available regarding the effects of exosomes in the regeneration of dental pulp *in situ*. A previous study that utilized a tooth root slice model that was implanted subcutaneously in the back of athymic nude mice to test *in vivo* dental pulp regeneration 105. The results of this study indicated that exosomes isolated either from normal pulp cells or odontogenic pulp cells can trigger the regeneration of dental pulp-like tissue, and the latter shows better regenerative efficiency than the former.

Unlike the numerous therapeutic applicaitions of stem cells, *in vivo* data on the use of exosomes as potent cell-free therapeutic agent in the field of periodontal and dental diseases are scarce. Research on exosome applications for periodontal and dental regeneration is still in its early stage. Further research on exosomes, especially *in vivo* applications that include animal models and clinical trials, should be conducted to harness the potential of exosomes as therapeutics agents.

### Skin and wound healing

**Figure 4:**
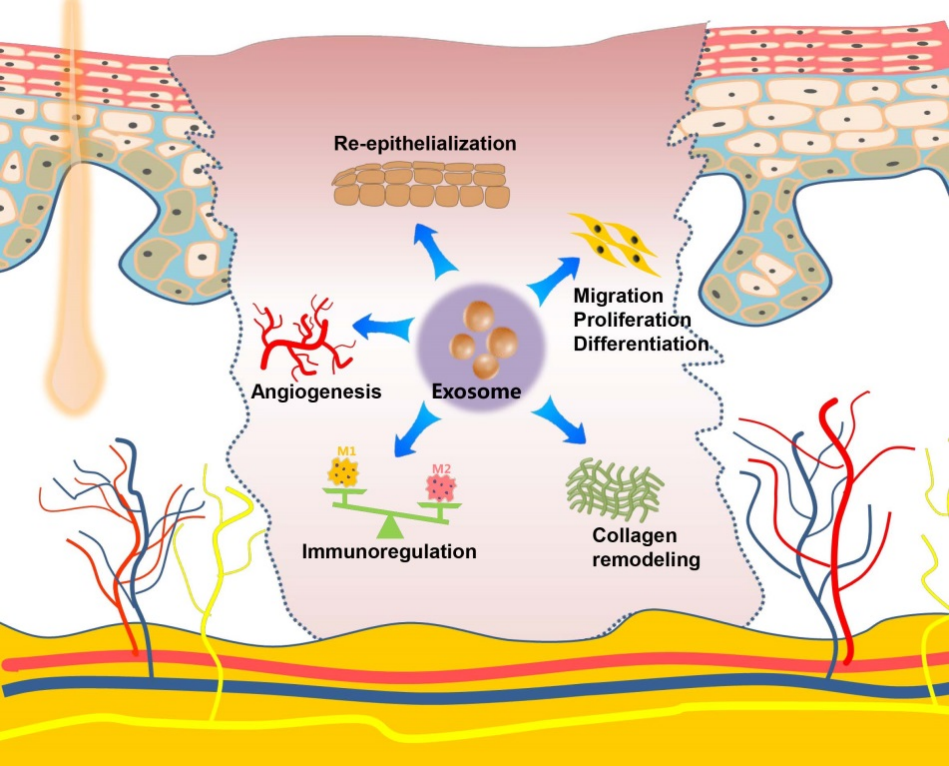
** Role and regulatory mechanism of exosomes on the skin and wound healing.** Exosomes exert their repair capacity in skin injury by promoting skin cell migration, proliferation and differentiation, enhancing re-epithelization, stimulating angiogenesis, remodeling collagen, and regulating the immune function.

The skin is the largest organ in the human body and plays an important role in the defense against the invasion of pathogenic microorganisms. Skin damage, which is frequently caused by extensive burns, trauma, or diabetic ulcers, result in a broad spectrum of complications with functional and cosmetic repercussions. Numerous studies show that exosomes derived from MSCs (ADSCs, gingival MSCs, and umbilical cord-derived MSCs), incorporated into a hydrogel scaffold, have therapeutic effects when used as a potential cell-free therapeutic tool for *in situ* full-thickness cutaneous wound healing 106-110. Along with the scaffold-loading exosome strategies discussed above, local injection (subcutaneously and intradermally) 111, 112 or systemic administration, such as the intravenous injection 113 of MSCs-derived exosomes into the wound sites of skin, presents another commonly used strategy for evaluating the effects of exosomes on cutaneous wound healing. Researchers have also developed a gene-modified MSCs-derived exosome-based miRNA delivery strategy to enhance therapeutic efficacy 114, 115. The engineered exosomes exhibit excellent effects on accelerating diabetic wound healing by increasing re-epithelization and stimulation of angiogenesis. Except for MSCs, bone marrow derived-macrophages, platelet-rich plasma (PRP), umbilical cord blood plasma, and oral mucosal epithelial cells are possible sources of exosomes for skin wound repair 116-119. Zhang *et al.* established a rat model of skin deep second-degree burn wound and demonstrated that the subcutaneous injection of human umbilical cord MSCs-derived exosomes significantly promotes cutaneous wound healing 120, 121. Although almost all of these studies used a skin defect or burn model on the back, these models are also applicable for craniofacial skin, given that the fundamental steps of the typical wound-healing process are conserved in the skins at different anatomical sites. Recently, a UVB-induced skin photoaging model was created and used to investigate the effects of exosomes on anti-ageing properties, in which exosomes derived from the three-dimensional human dermal fibroblast spheroids reduced skin ageing by regulating dermal fibroblast proliferation, migration, and protein expression effectively 122. Oral gingival/mucosal wounds, which mostly heal with minimal to no scarring similar to fetal wounds and heal faster than cutaneous wounds 123, have been studied to a lesser extent than cutaneous healing. A study involving a palatal gingival wound model indicated that exosomes secreted by gingival MSCs have therapeutic effects on wound healing 124. Overall, exosomes, mostly from stem cells, promote the regeneration of skin wounds by enhancing angiogenesis, stimulating the migration, proliferation, and differentiation, facilitating re-epithelialization and collagen remodeling, as well as regulating the immune activity. Exosome-mediated therapy could provide a multifaceted strategy for promoting cutaneous regeneration and repair (**Figure 4**).

### Oral cancer treatment

Head and neck cancer, with oral carcinoma as its major subtype, is one of the most widespread malignancies worldwide. Despite the continuous progress in its treatment and diagnosis, the 5-year overall survival rate of oral cancer remains low at approximately 50% 125, 126. Growing evidence demonstrates that exosomes shuttle different agents, including small interfering RNAs (siRNAs), miRNAs, and targeted drugs as therapeutic molecules into recipient cells, thereby attenuating the bioactivity for OSCC cells 127-130. A previous review discussed and summarized various aspects of exosome biology and functions in head and neck squamous cell carcinoma well 12. In contrast to studies involving animal testing and certain clinical trials on exosome application as treatment agents for other cancer types, preclinical studies involving *in vivo* exosome-based therapy for oral cancer are limited. A recent study involving the use of the hamster buccal pouch carcinoma model, a preclinical model that closely mimics human OSCC, assessed the effects of exosome treatment on oral cancer in live animals and showed that the antitumor effect of the intra-tumoral injection of stem cell exosomes is associated with the loss of tumor vasculature 131. As mentioned earlier, stem cell-derived exosomes have been shown to promote angiogenesis in tissue regeneration. However, in tumor treatment, they present as anti-angiogenic agents. The underlying mechanisms responsible for the pro- or anti-angiogenic property of stem cell exosomes remain unclear. This could possibly be attributed to receptor-mediated specific molecules intercellular transportation. Exosome-based oral cancer therapy remains at its infancy in contrast to its considerable application in oral cancer diagnostic. More studies using relevant preclinical models are required to validate the potential value of exosome in oral cancer treatment.

### OPMD treatment

OPMD includes oral leukoplakia, erythroplakia and oral submucous fibrosis, and its malignant transformation into oral cancer is highly associated with chronic inflammation 24. MSCs-derived exosomes and exosomal miR-8485 have been proved to be involved in premalignant lesions and carcinogenesis, indicating that intervention with the secretion of MSCs-derived exosomes could be an innovative strategy to prevent carcinogenesis 132. Wang *et al.* applied the MSCs-derived exosomes on buccal lesions in a dimethylbenzanthracene (DMBA)-induced OPMD model and demonstrated the feasibility of exosome-carried miR-185 as a novel therapeutic option for treating OPMD 133. Numerous treatments have been recommended for OPMDs, ranging from medical and surgical interventions, lasers, and photodynamic therapy 134. Multicenter randomized clinical trials with larger sample sizes should be conducted to ascertain whether exosome therapies have an advantage over these traditional remedies.

### Others

In addition to its application in commonly occurring diseases discussed above, exosome-based therapy has been utilized in relatively rare diseases in the craniofacial and dental domain. Investigators reported that SHEDs-derived exosome could be administered to manage traumatic brain injury 135. Most interestingly, Zhang *et al.* used a critical-sized tongue defect model in rats and showed that combinatory transplantation of small intestinal submucosa-extracellular matrix with gingival mesenchymal stem cells-derived exosomes promotes tongue lingual papilla recovery and taste bud regeneration 136. These studies provide more insights on exosome-based therapeutic applications in craniofacial and dental diseases and show that exosome-based strategies are not merely applicable in existing demonstrations.

**Figure 5:**
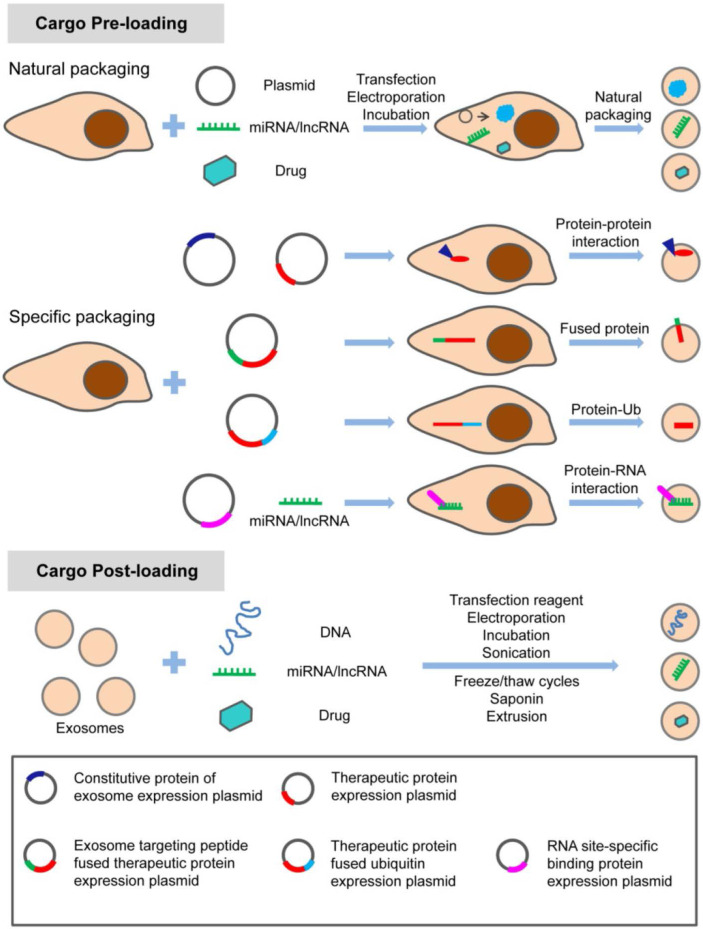
** Modification strategy for loading therapeutic molecules into exosomes.** The specific treating molecules (proteins, nucleic acids, and small chemicals) can be loaded into exosomes before or after exosome production through natural or specific packaging. Adapted with permission from 54, copyright 2019 Ivyspring International Publisher.

In summary, exosome-based therapy has great application potential from regenerative medicine to oncology in the craniofacial and dental field. As shown in **Table 3**, natural exosomes were utilized in a considerable number of studies, with few studies adopting engineered exosomes loading specific nucleic acids. Strategies with specific modifications maximize the therapeutic potential of exosomes in the craniofacial and dental fields. Different options are considered to meet this purpose. There are various modification methods for loading the specific treating molecules (proteins, nucleic acids, and small molecule drugs) into exosomes (**Figure 5**). Apart from loading, targeting strategy is also a potential enhancer for the therapeutic application of exosomes. Targeting exosomes could be acquired by the assembly of specific ligands on the exosome surface that recognize the target receptor of recipient cells. For instance, regarding the pro- or anti-angiogenic characteristic of stem cells, a novel and powerful engineered exosome with anti-tumor effect could be constructed by loading it with anti-angiogenic proteins, miRNAs, and equipping it with iRGD peptide (targeting tumor cells) on the surface. The recent emergence of potential exosome-mimetics with similar structure and biomarkers of exosomes and the ability to overcome drawbacks, such as low loading efficiency and low production yields, significantly promotes the development of conventional exosome-based therapy 137, 138. In our recent study, we fabricated a specific exosome-mimetics by serial mechanical extrusion and encapsulated it with the plasmid gene of vascular endothelial growth factor. Then the engineered exosome-mimetics was integrate into a biotin-avidin modified coaxial electrospun through covalent bonding. This well-designed, functional exosome-mimetics-mediated compound sustainably delivers the VEGF gene and significantly enhances osteogenesis and angiogenesis in a cranial defect model 139. The potential therapeutic effects of exosomes provide a great opportunity for developing exosome-related biomedical applications in various fields along with chemical, cellular, and genetic engineering techniques.

## Challenges and Perspectives

Considerable progress in the field of exosomes has increased our understanding of their biogenesis, molecular content, and biological function over the last decade. Exosomes have received considerable research attention as mediators of intercellular communication given their potential role as biomarkers and therapeutics. Using exosomes as biomarkers and therapeutic agents in the clinical application has several advantages. Exosomes-based diagnosis has minimal trauma and wide availability in various bodily fluids. The diversity of exosomal cargos, which provides multiple diagnostic parameters, could enhance the diagnostic sensitivity and specificity. The encapsulated analyte is protected from degradation, and it is stable due to the exosome protection by their bilayer lipid membrane. Regarding therapeutic applications, exosomes have been shown relatively free of ethical issues, excellent immune-compatibility, lower toxicity, efficient cellular entry, intrinsic ability to traverse biological barriers, and potential targeting ability through the surface-specific domain. Besides, since exosomes house multiple biomolecule types, they exert different therapeutic mechanisms simultaneously. Furthermore, exosomes can be modified, including internal loading or surface modification, due to their unique structure and physicochemical characteristics.

Despite the advances discussed herein, there are numerous limitations and challenges to be overcome before exosomes could be translated successfully into clinical applications. This extends beyond the craniofacial and dental field to the entire biomedical field. The major challenge prohibiting exploring exosomes in clinical applications is the lack of reliable and standardized methods for large-scale production and distinguishing them from other EVs. This is the first critical issue that requires to be addressed. Various separation strategies, including ultra-speed centrifugation, ultrafiltration, immunoaffinity capture and charge neutralization-based polymer precipitation, have been reported 140. However, it is difficult to identify which isolation and purification strategy produce optimum results. We speculate that ultracentrifugation is as one of the most common methods for exosome isolation before the emergence of high specific markers for exosome. Besides, exosomes are cell-derived vesicles that contain distinct bioactive cargos and present in the conditioned media of cultured cells and almost all bodily fluids. Considerable attention must be accorded to the stability and storage strategies necessary to translate the striking preclinical consequences of exosomes into clinical and commercial success as off-the-shelf diagnostic and therapeutic tools. Cryopreservation methods have drawn growing interest in exosome storage. However, controversy exists on whether the freeze-thaw cycles affect the stability of exosomes 141. Therefore, further preclinical testing should be designed to develop novel preservation strategies tailored for exosomes before clinical applications on a large scale. Additionally, there are controversies regarding defining exosome dosage, *i.e*., the concentration of exosomal proteins or the number of particles. This optimization is particularly crucial for clinical trials and requires relevant criteria to be proposed by reputable industry associations. Moreover, an appropriate cell source for exosomes based on their intended therapeutic use is essential. At present, the well-studied stem cells are widely used in certain diseases owing to their inherent attributes. The list of additional cell sources that require further characterization and detection continues to grow.

Despite the current challenges, the idea of using exosomes as a diagnostic and therapeutic tool is promising and inspiring. It is highly expected that, with adequate research, the additional exciting applications of exosomes in clinical practice are expected in the near future.

## Figures and Tables

**Table 1:** Exosome biomarkers for craniofacial and dental diseases

Disease	Biofluid	Biomarker	Type	Methods	Ref
**Periodontitis**	Saliva	CD9, CD81	Protein	ELISA	16
	Saliva	CA2, Histone H2A type 1, Ig kappa chain V-I region AU, HBG1, HIST1H2BJ HIST1H1D, CCL28, HIST1H1B, CPN2, C8A, PGLYRP2, SAA1, C1S, SELL, Ig kappa chain V-II region FR, BLVRB, C6, BPGM, CPB2, Ig heavy chain V-III region KOL, EHD2, Ig lambda chain V-II region TRO, MDK, APOL1, C8B, Ig lambda chain V-I region VOR	Protein	LC-MS/MS	17
	Saliva	PD-L1	mRNA	qRT-PCR	20
	Saliva	hsa-miR-140-5p, hsa-miR-146a-5p, hsa-miR-628-5p	microRNA	qRT-PCR	21
**OLP**	Saliva	miR-4484	microRNA	MicroRNA microarray analysis, qRT-PCR	26
	Plasma	miR-34a-5p	microRNA	MicroRNA microarray analysis, qRT-PCR	27
**Oral cancer**	Oral fluid	CD81, CD9, CD63, total concentration, size	Protein	AFM, ELISA	31
	Saliva	CD63, populations, morphologies, size	—	AFM	32
	Saliva	Spectral signature	—	FTIR, machine learning	33
	Plasma	CD63, CAV-1	Protein	Immunocapture-based analysis	34
	Plasma	PF4V1, CXCL7, F13A1, ApoA1	Protein	LC-MS/MS	35
	Plasma	PD-L1	Protein	Flow cytometry	36
	Plasma	Laminin-332	Protein	ELISA	37
	Saliva	mucin 5B, galectin-3-binding protein, immunoglobulin alpha-1 chain c region, prolactininducible protein, alpha-2-macroglobulin, haptoglobin alpha chain, pyruvate kinase isozymes M1/M2, glyceraldehyde-3-phosphate dehydrogenase	Protein	LC-MS/MS	38
	Plasma	hsa-miR-19a, hsa-miR-512-3p, hsa-miR-27b, hsa-miR-20a, hsa-miR-28-3p, hsa-miR-200c, hsa-miR-151-3p, hsa-miR-223, hsa-miR-20b, hsa-miR-22, hsa-miR-516-3p, hsa-miR-370, hsa-miR-139-5p, hsa-let-7e, hsa-miR-145-3p, hsa-miR-30c	microRNA	MicroRNA microarray analysis, qRT-PCR	39
	Saliva	miR-302b-3p, miR-517b-3p, miR-512-3p, miR-412-3p	microRNA	MicroRNA microarray analysis	40
	Saliva	miR-24-3p	microRNA	MicroRNA microarray analysis	41
**Sjögren's syndrome**	Saliva	hsa-miR-150, hsa-miR-23a, hsa-miR-27b, hsa-miR-29b, hsa-miR-29c, hsa-miR-335, hsa-miR-379, hsa-miR-433, hsa-miR-454, hsa-miR-483-3p, hsa-miR-584, hsa-miR-621, hsa-miR-652, hsa-miR-760, hsa-miR-888, miRPIus_17824, miRPIus_17841, miRPIus_17848, miRPIus_17858	microRNAs	MicroRNA microarray analysis	44
	Saliva	HLA, S100A9, HLA-B, CAT, LY6D, TYROBP, DEFA1, RHOA, GNA13, SLPI, ANXA6, SIRPA, NCF1B, WDR1, MUC5AC, ARPC1B, LSP1, LV302, CD59, FCER1G, RP2, LCN2, HVCN1, KV312, GPI, PTPRJ, HLA-A, SNAP23, HV102, BASP1, APMAP, GNAI3, RAB5C, ICAM3, CA4, MUC2, HIDE, PTPRC, SLC6A14, ACTN1, PPP1CA, CD9, SPRR1A, CALM1,FERMT3, BSG, CNP, UBA52, TLN1, CAPZB, CORO1A, DSTN, SLC9A3R2, KV303, SIGLEC5, OLFM4, GNB2 ARRB2, PGLYRP1, NCF2	Protein	LC-MS	45
	Tear	CPNE1, CALM	Protein	LC-MS	45

LC‐MS/MS: liquid chromatography-tandem mass spectrometry; ELISA,:enzyme-linked immunosorbent assay; OLP: oral lichen planus; AFM: atomic force microscopy; FTIR: Fourier transform infrared spectroscopy; LC-MS: liquid chromatography-mass spectrometry.

**Table 2:** Oral exosome biomarkers for the detection of systemic diseases

Systemic Disease	Oral Biofluid	Biomarker	Type	Methods	Ref
Inflammatory bowel disease	Saliva	PSMA7	Protein	LC-MS/MS	46
Ageing	Saliva	miR-24-3p	microRNAs	MicroRNA microarray analysis	47
Pancreatic cancer	Saliva	Apbb1ip, Daf2, Foxp1, Incenp, Aspn, BC031781, Gng2	mRNA	Microarray	48
Pancreatobiliary tract cancer	Saliva	miR-1246, miR-4644	microRNAs	qRT-PCR	49
Lung cancer	Saliva	Annexin Al, A2, A3, A5, A6, A11, NPRL2, CEACAM1, HIST1H4A, MUC1, PROM1, TNFAIP3	Protein	LC-MS/MS	50
Lung cancer	Saliva	BPIFA1, CRNN, MUC5B, IQGAP	Protein	LC-MS/MS	51
Melanoma	Saliva	Human melan-A	RNA	qRT-PCR	52
Gestational diabetes mellitus	Gingival crevicular fluid	Total concentration	—	Qubit protein assay kit	53

LC‐MS/MS: liquid chromatography-tandem mass spectrometry.

**Table 3:** Summary of *in vivo* studies on exosome-based therapy in craniofacial and dental application

Tissue	Model/Species	Exosome Origin	Involved Pathway	Administration Methods	Functional Effects	Ref
Bone regeneration	Calvarial bone defect/ Osteoporotic rats	hiPSC-MSCs	—	β-TCP scaffold	Promote osteogenesis and angiogenesis	60
	Calvarial bone defect/ Mice	hADSCs	—	PLGA scaffold	Promote osteogenesis	62
	Calvarial bone defect/ Rats	hiPSC-MSCs	PI3K/Akt	β-TCP scaffold	Promote osteogenesis	81
	Calvarial bone defect/ Rats	miR-375- overexpressed hADSCs	—	Hydrogel	Promote osteogenesis	61
	Calvarial bone defect/ Rats	DMOG-stimulated hBMSCs	AKT/mTOR	Porous HA scaffold	Stimulate angiogenesis	67
	Calvarial bone defect/ Mice	hPSCs	SAPK/JNK, HGF, Sirtuin, FGF, PDGF, AMPK, PTEN	Percutaneous injection	Promote osteogenesis	63
	Alveolar bone defect/ Rats	SHEDs	AMPK	β-TCP scaffold	Promote osteogenesis and angiogenesis	82
	BRONJ/Rats	hBMMSCs	—	Tail vein injection	Promote angiogenesis and bone regeneration	83
TMJ regeneration	TMJ osteoarthritis/Rats	hESC-MSCs	AKT, ERK, AMPK	Intra-articular injection	Suppress inflammation and pain , reduce apoptosis, enhance matrix synthesis	88
Periodontal regeneration	Periodontitis/Rat	rADSCs	—	Pocket local injection	Anti-inflammatory effect, enhance osteoid tissues and blood vessels formation	93
	Periodontal intrabony defects/Rats	hESC-MSCs	AKT, ERK	Collagen sponge	Enhance osteogenesis and periodontal ligament formation	94
	Periodontitis/Human clinical trial	hADSCs	—	Local injection into periodontal pockets	—	NCT04270006
Dental pulp regeneration	Tooth root slice model/Mice	hDPSCs under odontogenic conditions	P38 MAPK	Collagen membrane	Promote stem cell differentiation and blood vessels formation	105
Skin regeneration	Full-thickness skin wounds/ Diabetic mice	mADSCs	—	FEP hydrogel	Stimulate angiogenesis and enhance cell proliferation, granular tissue formation, collagen deposition, remodeling and re-epithelialization	106
	Full-thickness skin wounds/ Diabetic mice	mADSCs	—	FHE hydrogel	Promote angiogenesis, re- epithelialization and collagen deposition	107
	Full-thickness skin wounds/ Rat	rADSCs	—	Alginate hydrogel	Enhance re-epithelialization, collagen deposition and angiogenesis	108
	Full-thickness skin wounds/ Diabetic rats	hGMSCs	—	Chitosan/silk hydrogel sponge	Promote re-epithelialization, deposition and ECM remodeling and enhance angiogenesis and neuronal ingrowth	109
	Full-thickness skin wounds/ Mice	HucMSCs	TGF-β/SMAD2	HydroMatrix hydrogel	Suppress myofibroblast aggregation and scar formation	110
	Full-thickness skin wounds/ Mice	hADSCs	PI3K/Akt	subcutaneous and intradermal injection	Promote collagen synthesis and optimize collagen deposition	111
	Full-thickness skin wounds/ Mice	hMenSCs	NF- kB	Intradermal injection	Induce M1-M2 macrophage polarization, enhance neoangiogenesis and re-epithelialization	112
	Full-thickness skin wounds/ Mice	hBMMSCs	—	Intravenous injections	Promote macrophages towards M2 polarization	113
	Full-thickness skin wounds/ Diabetic mice	miR-21-5p overexpressed hADSCs	Wnt/β-catenin	Direct addition, coverage with alginate gel	Enhance re-epithelization, collagen remodeling, angiogenesis and vessel maturation	114
	Full-thickness skin wounds/Diabetic mice	mmu_circ_0000250 overexpressed mADSCs	miR-128-3p/SIRT1	Subcutaneous injection	Promote angiopoiesis and suppress apoptosis by autophagy	115
	Full-thickness skin wounds/ Diabetic rat	hPRP	RhoA/YAP, PI3K/Akt, Erk1/2	Sodium alginate hydrogel	Promote angiogenesis	116
	Skin wound model/ Mice	mM2 Mφs	—	Subcutaneous injection	Enhance angiogenesis, re-epithelialization, and collagen deposition	117
	Full-thickness skin wounds/ Rats	hOMECs	—	Direct addition, coverage with TegaDerm and gauze	Reduce fibroblast proliferation and stimulate the release of growth factors	118
	Full-thickness skin wounds/ Mice	hUCB	PTEN, SPRY1	Subcutaneous injection	Promote re-epithelization and angiogenesis	119
	Deep second-degree burn wound/ Rats	HucMSCs	Yap, Wnt/β-Catenin	Subcutaneous injection	Restrict stem cell expansion and collagen deposition	120
	Deep second-degree burn wound/ Rats	HucMSCs	Wnt/β-Catenin, AKT	Subcutaneous injection	Enhance proliferation of skin cells and promote re-epithelialization	121
	UVB-Induced skin photoaging/ Mice	HDFs	TGF-β	Dermo-jet model G (needleless injection)	Regulate dermal fibroblasts to induce efficient collagen biosynthesis and ameliorate inflammation	122
	Full-thickness gingival wound/ Mice	mGMSCs	Fas/Fap-1/Cav-1	Submucosally injection	Produce high amounts of IL-1RA	124
Oral Cancer	DMBA-induced OSCC/ Hamsters	hMenSCs	—	Injection into the base of the tumor	Reduce tumor vasculature	131
OPMD	DMBA-induced OPMD/ Hamsters	microRNA-185 overexpressed mBM-MSCs	Akt	Solution painting	Alleviate inflammation, inhibit cell proliferation and angiogenesis and induce apoptosis	133
Others	Free-falling induced TBI/ Rats	SHEDs	—	Injection into rat brains	Shift microglia M1/M2 polarization	135
	Critical-sized tongue defect/ Rats	hGMSCs	—	SIS-ECM	Promote reepithelialization, reinnervation and taste bud regeneration	136

hiPSC-MSC: human induced pluripotent stem cells-derived mesenchymal stem cells; β-TCP: β-tricalcium phosphate; hADSCs: human adipose-derived mesenchymal stem cells; PLGA: poly(lactic-co-glycolic acid); DMOG: dimethyloxaloylglycine; hBMSCs: human bone mesenchymal stem cells; HA: hydroxyapatite; hPSCs: human perivascular stem cells; SHEDs: stem cells from human exfoliated deciduous teeth; TMJ: temporomandibular joint; hESC-MSCs: human embryonic stem cell-derived MSCs; rADSCs: rat adipose-derived stem/stromal cells; hDPSCs: human dental pulp stem cells; mADSCs: mice adipose-derived mesenchymal stem cells; FEP: F127 (F127) grafting polyethylenimine (PEI) and aldehyde pullulan (APu); FHE: multifunctional hydrogel composed of Pluronic F127, oxidative hyaluronic acid and Poly-ε-L-lysine; hGMSCs: human gingival mesenchymal stem cells; ECM: extracellular matrix; HucMSCs: human umbilical cord mesenchymal stem cells; hMenSCs: human menstrual blood-derived mesenchymal stem cells; hBMMSCs: human bone marrow-derived mesenchymal stem cells; hPRP: human platelet-rich plasma; mM2 Mφs: mice M2 phenotype macrophages; hOMECs: human oral mucosa epithelial cells; hUCB: human umbilical cord blood; HDFs: human dermal fibroblasts; mGMSCs: mice gingiva-derived mesenchymal stem cells; DMBA: dimethylbenzanthracene; OPMD: oral potentially malignant disorders; mBM-MSCs: mice bone marrow-derived mesenchymal stem cells; TBI: traumatic brain injury; BRONJ: bisphosphonate-related osteonecrosis of the jaw; SIS-ECM: small intestinal submucosa-extracellular matrix.
